# Characterization of a Bayesian genetic clustering algorithm based on a Dirichlet process prior and comparison among Bayesian clustering methods

**DOI:** 10.1186/1471-2105-12-263

**Published:** 2011-06-28

**Authors:** Akio Onogi, Masanobu Nurimoto, Mitsuo Morita

**Affiliations:** 1 Maebashi Institute of Animal Science, Livestock Improvement Association of Japan, Inc., 316 Kanamaru, Maebashi, Gunma, 371-0121, Japan

## Abstract

**Background:**

A Bayesian approach based on a Dirichlet process (DP) prior is useful for inferring genetic population structures because it can infer the number of populations and the assignment of individuals simultaneously. However, the properties of the DP prior method are not well understood, and therefore, the use of this method is relatively uncommon. We characterized the DP prior method to increase its practical use.

**Results:**

First, we evaluated the usefulness of the sequentially-allocated merge-split (SAMS) sampler, which is a technique for improving the mixing of Markov chain Monte Carlo algorithms. Although this sampler has been implemented in a preceding program, HWLER, its effectiveness has not been investigated. We showed that this sampler was effective for population structure analysis. Implementation of this sampler was useful with regard to the accuracy of inference and computational time. Second, we examined the effect of a hyperparameter for the prior distribution of allele frequencies and showed that the specification of this parameter was important and could be resolved by considering the parameter as a variable. Third, we compared the DP prior method with other Bayesian clustering methods and showed that the DP prior method was suitable for data sets with unbalanced sample sizes among populations. In contrast, although current popular algorithms for population structure analysis, such as those implemented in STRUCTURE, were suitable for data sets with uniform sample sizes, inferences with these algorithms for unbalanced sample sizes tended to be less accurate than those with the DP prior method.

**Conclusions:**

The clustering method based on the DP prior was found to be useful because it can infer the number of populations and simultaneously assign individuals into populations, and it is suitable for data sets with unbalanced sample sizes among populations. Here we presented a novel program, DPART, that implements the SAMS sampler and can consider the hyperparameter for the prior distribution of allele frequencies to be a variable.

## Background

In population genetics, inference of population structures is important for various purposes such as assessment of genetic diversity, detection of genetic discontinuities in natural wildlife habitats, and correction for stratification in association studies. To infer population structures without prior knowledge about the population, various statistical approaches using neutral molecular markers have been proposed [1-9].

Bayesian approaches using Markov chain Monte Carlo (MCMC) methods have been widely used to infer population structures since Pritchard *et al. *[1] proposed the Bayesian clustering algorithms implemented in the well-known program STRUCTURE. This program can infer the assignment of individuals to populations or the admixture proportions of individuals for a given number of populations (K). Researchers have extended Bayesian algorithms for various purposes such as to take advantage of spatial information [10-14], estimate inbreeding coefficients [15], allow for allele mutations [16], and infer K values [10,17-19].

Pella and Masuda [18] used a Dirichlet process (DP) to infer K values. DP is a stochastic process that was proposed by Ferguson [20] to treat nonparametric problems in Bayesian frameworks. The merit of using DP to infer K is that K can take any value between 1 and the number of individuals (i.e., the maximum value for K), and thus, few assumptions about K are needed for inference. Pella and Masuda [18] considered K and the assignment of individuals to populations as random variables using DP as a prior distribution for K and allele frequencies unique to populations. Huelsenbeck and Andolfatto [19] also used the DP prior for the inference of population structures, and Reich and Bondell [14] proposed a clustering algorithm using the DP prior, which can incorporate spatial information. Besides the inference of population structures, DP priors have been used to infer the number of ancestral haplotype blocks [21], to model nonsynonymous/synonymous rate ratios [22], and to model the selfing rates of individuals [15].

To date, two clustering programs that implement the DP prior have been provided, HWLER [18] and STRUCTURAMA [19]. Both programs implement the Gibbs sampling procedure to infer the posterior distribution. These programs differ in their approach to improve the mixing of MCMC algorithms. HWLER implements the sequentially-allocated merge-split (SAMS) sampler, which moves multiple observations simultaneously [23], and STRUCTURAMA implements the Metropolis-Coupled MCMC (MCMCMC) technique [24], which runs multiple chains, some of which are closer to a uniform distribution than the target distribution, and attempts to swap states among chains.

Although HWLER and STRUCTURAMA are useful and have been used in some recent studies [25-30], their application to real data sets has been less common compared with that of STRUCTURE. This may be because the properties of these methods have not been investigated in detail. When results obtained with different methods are inconsistent, it is difficult to interpret the results. For example, HWLER and STRUCTURAMA may provide inconsistent results. In addition, although HWLER detects three populations, the result obtained with STRUCTURE under the assumption that K is 3 may be different from that obtained with HWLER.

The purpose of this study was to characterize the Bayesian method based on the DP prior and to provide information that will be useful in practice. First, we evaluated the SAMS sampler because its effectiveness has not been examined. Second, we investigated the effect of a hyperparameter, named *λ*, which defines the prior distribution of allele frequencies, on the performance of this method. As described by Pella and Masuda [18], HWLER set *λ *to  where *J*_*l *_is the number of alleles at locus *l *for any loci. However, this specification resulted in inaccurate inferences in some cases. Third, we compared the DP prior method with other Bayesian methods that infer the assignment of individuals for a given K value. We focused primarily on the effect of unbalanced sample sizes among populations on the behavior of these methods because unbalanced sample sizes were found to affect the behavior of these methods in our preliminary study.

## Methods

### Assumption and goal

We assumed that a sampled data set consists of diploid individuals derived from an unknown number of populations, which are defined by unique values of allele frequencies. Although our novel program, named DPART, can also analyze haploid populations, we assumed only diploid individuals in this study for simplicity. Our goal was to infer the number of populations and assign individuals to their populations based on their genotypes. The estimation of admixture proportions was not of interest.

### DP prior

The mathematical details of DP can be found in studies conducted by Ferguson [20], Antoniak [31], and Neal [32]. Here we provided a brief description of the DP prior. Approximately and intuitively, DP can be seen as a stochastic process that converts a continuous distribution to a discrete distribution. We assumed that observations (genotypes) are generated from the following model (for simplicity, in this paragraph, we considered a situation where only one locus is involved). Let *G*_0 _denote the prior distribution of allele frequencies at the locus, which is continuous. DP is defined by *G*_0 _and the concentration parameter *α *(>0). DP divides *G*_0 _into a number of classes, each of which is represented by a single point and yields a discrete probability distribution, *G*. The number of classes, which is determined by the number of observations and *α*, can be infinite. A vector of allele frequencies (i.e., frequencies for all alleles at the locus) for each genotype is drawn from *G *instead of *G*_0_, and genotypes are drawn from the corresponding allele frequency vectors. This model can be written as follows:(1)

where *x*_*i *_is the genotype of individual *i*, *ϕ*_*i *_is the allele frequency vector for genotype *i*, and *DP *(*G*_0_, *α*) is DP. This model is known as the DP mixture model [31]. Because *G *is discrete, allele frequency vectors for some genotypes may have values in common, i.e., these genotypes can be seen as members of the same population, which is characterized by the shared allele frequency vectors.

### Parameters

By integrating out *G*, we can obtain a simpler representation of the model. When *G *is integrated out, the predictive (posterior) distribution for the allele frequency vector *ϕ*_*i *_conditional on {*ϕ*_1_, ..., *ϕ*_*i*__-1_} can be written as follows:(2)

where *n*_*j *_is the number of allele frequency vectors that share values with *ϕ*_*j*_. This sequence of predictive distributions is known as a Polya urn scheme [33]. To represent the clustering property of the model more explicitly, we let {*ϕ*_1_,...,*ϕ*_*k*_} denote allele frequency vectors unique to populations {1,...,*K*}. In addition, we introduced parameters that represent the partition of individuals {1,...,*n*}, according to the parameterization of Dahl [23]. We let *η *= {*S*_1_,...,*S*_*K*_} define a partition for {1,...,*n*} such that , *S*_*i *_∩ *S*_*j *_= ∅ for all *i *≠ *j*, and *S*_*i *_≠ ∅ for all *i*.

Eq. (2) includes the following two types of prior information:(3)

where *η*^<^^*i *^is *η *before assigning the *i*th individual and |*S*_*j*_| is the number of individuals included in *S*_*j*_, which is the prior for the number of populations (K) and assignment of individuals, and

which is the prior for allele frequencies for each population. Now, we can rewrite Eq. (1) as follows:

where *X *= {*X*_1_,...,*X*_*n*_} is a vector of the genotypes of the individuals {1,...,*n*}, *I*{*i *∈ *S*_*j*_} = 1 if *i *∈ *S*_*j*_, and otherwise 0, and(4)

where Γ is the gamma function. Note that Eq. (4) results from the products of Eq. (3).

### Integration of allele frequencies

Although some clustering methods infer allele frequencies (e.g., STRUCTURE), allele frequencies can be analytically integrated out as in HWLER and STRUCTURAMA. Thus, our goal is to infer not *f *(*η*, *ϕ *| *X*) but *f *(*η *| *X*). By Bayes' theorem, this can be written as follows:

where(5)

where  denotes the genotypes of individuals included in *S*_*j *_and  is the posterior distribution of *ϕ*_*j *_updated from *G*_0 _on the basis of the genotypes of individuals assigned to *S*_*j *_preceding the *i*th individual. *G*_0 _is assumed to be a flat Dirichlet distribution. The prior probability density of *ϕ *is given as follows:

where *L *is the number of loci, *J_l _*is the number of alleles at locus *l*, *λ_l _*is the hyperparameter for locus *l*, and *ϕ*_*jlh *_is the frequency of allele *h *at locus *l *of population *j*. Rannala and Mountain [34] provided the following equation:

where *y*_*jlh *_is the number of copies of allele *h *at locus *l *in individuals assigned to *S*_*j *_preceding the *i*th individual and *y*_*jl *_= ∑*y*_*jlh*_. *L*(*X*_*i*_|*ϕ*) in Eq. (5) can be rewritten as follows:

where *δ*(*x*_*il*_) = 1, if the genotype of individual *i *at locus *l *is heterozygous and if not is 0, and *n*_*ilh *_is the number of copies of allele *h *at locus *l *in individual *i*. Now the integral in Eq. (5) can be solved analytically (for an example, see [19] or [34]) as follows:

### Gibbs sampler

Neal [35] and MacEachern [36] introduced the Gibbs sampling procedure, which integrates out model parameters (here *ϕ*). Let *η*^-^^*i *^denote *η *when only the *i*th individual is removed. The conditional probability of the assignment of individual *i *is given as follows:(6)

where *b *is the normalizing constant. Because individuals are exchangeable, one can treat every individual as the last observation. The integrals in Eq. (6) can be solved analytically. The details of Gibbs sampling is described in Endnote A. In our experiments, one MCMC iteration consisted of one scan of the Gibbs sampler. After burn-in, *η *is sampled in every predefined number of MCMC iterations.

### SAMS sampler

Because the Gibbs sampler moves only one observation, a large change hardly ever occurs, and tends to stay at a local mode. A remedy is to move multiple observations simultaneously. Jain and Neal [37] proposed a split-merge sampler, which proposes a new partition by splitting or merging components and accepts it with Metropolis-Hastings (MH) probability. When it splits a component, the proposed assignment of observations in the component is generated by repeating Gibbs sampling only for these observations. This method can produce more acceptable proposals than the random assignment of observations. Dahl [23] improved the method in terms of efficiency and proposed an alternative sampler, the SAMS sampler, which generates proposals by one cycle of allocation of observations instead of repeating Gibbs sampling. The algorithm of the SAMS sampling is described in Endnote B. In this study, the SAMS sampling was performed once in an MCMC iteration. Four iterations with the SAMS sampler were followed by one iteration with the Gibbs sampler. This cycle was repeated until the end of the iterations.

### Inference of the hyperparameter

*λ*_*l*_, which is the hyperparameter for the prior distribution of allele frequencies at locus *l*, can be inferred by the MH method. The joint posterior probability of *η *and *λ *= {*λ*_1_,...,*λ*_*L*_} can be written as follows:

When the prior distribution for *λ*_*l *_is assumed to be uniform, equations in the Gibbs and SAMS samplers can be used without modification. In this study, the prior for *λ*_*l *_was assumed to be uniform, *U*(0,10). The proposal of *λ*_*l*_, *λ*_*l*_', was generated from the normal distribution *N*(*λ*_*l*_, *δ*^2^), where *δ *is an arbitrary value, e.g., 0.02. The proposal was accepted with the probability:

where *λ*^-^^*l *^is *λ *when *λ*_*l *_is removed.

We can also assume that each locus shares a single value, *λ*_*a*_. In this case the probability becomes the following:

### Prior distribution of the number of populations

As seen in Eq. (3) or (4), the prior distribution of K depends on *α *and the number of individuals. We can infer this prior distribution by the Monte Carlo method (see Endnote C). For example, when n = 100 and *α *= 0.2, the expected K value is approximately 2.0. When *α *increases to 0.43, K increases to approximately 3.0. Although it is possible to infer *α *as well as *λ*, in this study, we fixed *α *through the MCMC iterations. The effect of *α *has been thoroughly examined by Huelsenbeck and Andolfatto [19]. The authors reported that the misspecification of *α *(i.e., the expected K value) could affect the results, especially when the number of loci was small.

### Summarizing the sampled partitions

Two methods were used to summarize the partitions that were sampled from the posterior distribution of *η*. For simulated data sets, we used the mean partition proposed by Huelsenbeck and Andolfatto [19], which is defined as follows:

where *v *is the number of sampled partitions and *d*(*η*_*i*_, *u*) is the partition distance between the sampled partition *η*_*i *_and mean partition. The partition distance between *η*_*i *_and *u *is defined as the minimum number of individuals that must be removed from both *η*_*i *_and *u *such that the two partitions are the same [38]. The partition distance was calculated using Eq. (16) of Konovalov *et al. *[39] to obtain the cost matrix, and we solved this using the Hungarian algorithm. The algorithm for calculating the mean partition is described in Endnote D. This statistic is useful for evaluating the posterior distribution of *η *automatically. We divided the partition distances by the number of individuals. Thus, the partition distances ranged from 0 to 1.

For real data sets, we performed agglomerative hierarchical clustering on the basis of co-assignment probabilities. This method was introduced by Dawson and Belkhir [2]. Briefly, after all samples were obtained, the co-assignment probabilities for all individual pairs were calculated from the sampled partitions. Next, complete linkage clustering was performed on the basis of the probabilities. We used the hclust function of the stats package of R for complete linkage clustering [40]. An example R code is provided in Additional file 1. Note that neither the mean partition nor hierarchical clustering are affected by the label-switching problem that often emerges in analyses using methods implementing the DP prior.

### Programs and MCMC parameters

We wrote the clustering programs in C. The program implementing the DP prior is referred to as DPART. In addition to DPART, we also produced two clustering programs that infer only the assignment of individuals under a given K value. These programs are based on algorithms proposed by Pritchard *et al*. [1] and Falush *et al*. [41]. In these algorithms, the prior probability that an individual belongs to each population is equal among the populations, and individuals are assigned to populations according to likelihood. These algorithms infer the allele frequencies of populations that are integrated out in DPART in addition to the assignment of individuals. One program assumes that there is no correlation among the allele frequencies of populations, and the other assumes a correlation. Thus, the former is equivalent to the "no admixture and no F model" setting of STRUCTURE and the latter is equivalent to the "no admixture and F model" setting. These programs are referred to as Fixed K and Uncorrelated Model (FUM) and Fixed K and Correlated Model (FCM), respectively. STRUCTURE infers admixture proportions of individuals until burn-in is half completed even when no admixture option is selected (see the source code of STRUCTURE). Because this setting helps the program to avoid the generation of an empty cluster, which has no individuals, we followed the setting. In FCM, the prior distribution of the drift parameter was assumed to be *U*(0,1). The programs provided in this study are summarized in Table 1. DPART is provided in Additional files 2, 3, 4, 5, 6 and 7. FUM and FCM can be reproduced by STRUCTURE.

**Table 1:** Programs provided in this study

Program	Purpose	Assumption	Precedent software equivalent to the program
DPART	Inference of K and assignment of individuals	K and assignment of individuals follow the DP prior	HWLER when *λ *is fixed to where *J*_*l *_is the number of alleles at locus *l*
FUM	Assignment of individuals	Allele frequencies of each population are drawn independently	STRUCTURE (no admixture and no F model)
FCM	Assignment of individuals	Allele frequencies of populations are correlated	STRUCTURE (no admixture and F model)

STRUCTURAMA was used for simulated data sets. The expected K value was set equal to the true K value. Four chains were run simultaneously and the temperature was set to 0.2. We also used STRUCTURE ver. 2.2 with its default setting, "admixture and F model," for the bull data set because it is a widely used method for the inference of population structures, although the estimation of admixture proportions was not of interest.

For comparison between DPART and STRUCTURAMA and examination of the effect of *λ*, the number of MCMC iterations was 20,000, and the first half of the iterations was discarded as burn-in. Sampling was performed every 10 iterations. For comparison among DPART, FUM, and FCM, the number of iterations was 100,000, and the first half of the iterations was discarded. Sampling was performed every 50 iterations. For the real data sets, the number of iterations was 500,000 and the first 400,000 iterations were discarded. Sampling was performed every 50 iterations.

### Simulation method 1

This simulation method was used to compare among clustering methods. EASYPOP was used to simulate populations under the Wright-Fisher model [42]. The island model was assumed. The number of populations was 8 and the number of individuals per population was 1,000. The number of generations, number of loci, mutation rate, and possible number of alleles were 20,000, 50, 0.0005, and 10, respectively, for microsatellites, and 5,000, 100, 5 × 10^-7^, and 2, respectively, for single nucleotide polymorphisms (SNPs). New alleles occurred in every possible allelic state with equal probability. Diploidy, random mating, one sex, and linkage equilibrium were also assumed. The genotypes of the first generation were generated by randomly assigning alleles from the possible allelic states. Migration rates were 0.005, 0.003, and 0.001 for microsatellites and 0.002 for SNPs. EASYPOP was run five times for each scenario. The resulting data sets are referred to as base population sets. Sampling was performed 20 times from each base population set to obtain 100 data sets per scenario. When two, three or four populations were sampled from the base population sets, base populations to be sampled were selected randomly. Similarly, when 10 or 20 loci were sampled, the loci to be sampled were also selected randomly. The pairwise *F*_*st *_values between the base populations for each migration rate are summarized in Table 2. Typically, the observed heterozygosities were approximately 0.81 for microsatellites and 0.36 for SNPs. The pairwise *F*_*st *_and heterozygosity values were calculated using GENETIX [43].

**Table 2:** Pairwise *F_st _*between base populations generated by simulation method 1

	Microsatellite			SNP
	
	M = 0.005	M = 0.003	M = 0.001	M = 0.002
Pairwise Fst	0.0371 (± 0.0036)	0.0610 (± 0.0049)	0.1298 (± 0.0105)	0.0996 (± 0.0079)

M indicates the migration rate.

### Simulation method 2

This method was used to investigate the effect of *λ*. The variance of a Dirichlet distribution, *Dirichlet *(*λ*,...,*λ*), decreases as *λ *increases. When allele frequencies are drawn from the Dirichlet distribution, it means that the frequencies closely approach uniformity among alleles as *λ *increases. Thus, it was expected that *λ *would affect clustering behavior depending on the uniformity of frequencies among alleles. To examine this hypothesis, we devised a simulation method on the basis of the allele frequency correlated model [41]. In this method, allele frequencies were generated from the Dirichlet distribution

where *Pa *is the allele frequency of a virtual ancestral population, *F *is the drift parameter, which is equivalent to Wright's *F*_*st*_, and *J*_*a *_is the number of ancestral alleles. We can determine any level of uniformity of frequencies among alleles by varying ancestral allele frequencies. We assumed two marker types, microsatellites (*J*_*a *_= 5) and SNPs (*J*_*a *_= 2). For microsatellites, the ancestral allele frequencies were {0.8,0.05,...,0.05} or {0.2,0.2,...,0.2} and *F *was 0.05, and for SNPs, the ancestral frequencies were {0.8,0.2} or {0.5,0.5} and *F *was 0.07. K was assumed to be 2 and the number of individuals was 25 per population. Genotypes were generated from the allele frequencies assuming random mating. If only one allele was observed at a locus, then that locus was excluded. One hundred data sets were generated for each scenario.

### Comparison among methods for simulated data sets

To compare methods for simulated data sets, we used the partition distance between the true and inferred partitions, which was denoted as . We calculated average  over the 100 simulated data sets and counted the number of data sets in which  was 0.1 or less. For DPART and STRUCTURAMA, we used the mean partition as the inferred partition and calculated the average K values in the mean partitions. When FUM and FCM were used, the inferred partition was determined on the basis of the probabilities of assigning individuals to populations, which were calculated from the sampled partitions. Although FUM and FCM may be evaluated with the mean partition, we used the partition that was based on the probabilities of assignment because this is computationally more feasible than the mean partition and label-switching occurs rarely in STRUCTURE-like algorithms as indicated by Pritchard *et al. *[1]. Both approaches provided almost the same partitions in our preliminary study.

Note that  can not be used for evaluation without modification when unbalanced sample sizes are present among populations. Suppose that two populations are included in a data set. If the sample sizes are uniform between the populations and an analysis fails to detect any population structures, i.e., all individuals are assigned to one cluster,  is 0.5. However, if the sample sizes are 10 and 300 and an analysis fails similarly,  decreases to 0.032 (10/310). Thus, in such cases, we calculated  as if an individual in the smaller subset was equivalent to 30 individuals in the larger subset. For example, if the sample sizes are 10 and 300 and only one individual in the smaller subset is incorrectly assigned to the larger subset,  is 0.05 (30/600) but not 0.003 (1/310).

### Chicken data set

This data set represents 600 chickens of 20 European breeds, which were genotyped for 27 microsatellites by Rosenberg *et al. *[44]. This data set was previously analyzed by Pella and Masuda [18] using HWLER. With only one run of HWLER, Pella and Masuda were able to obtain a result similar to that obtained by Rosenberg *et al. *with multiple runs of STRUCTURE. Here we used this data set to demonstrate the extent to which the choice of *λ *affects the behavior of the DP prior method.

### Bull data set

This real data set consists of 427 bulls maintained in Japan. These animals were born between 1984 and 2004 and had been genotyped for parentage testing. They included some half sibs but excluded full sibs. The number of microsatellites was 31 and the mean observed heterozygosity was 0.6463. This data set was used to demonstrate how unbalanced sample sizes among populations affect the behavior of clustering methods. This data set is provided in Additional file 8.

## Results

### Evaluation of the SAMS sampler

The SAMS sampler was implemented in HWLER to improve the mixing of MCMC algorithms [18]. However, the effectiveness of this sampler in population structure analysis was unknown. Therefore, we evaluated the SAMS sampler with our program, DPART, using simulated data sets generated by simulation method 1 and compared it with STRUCTURAMA. The number of populations (K) was 2, 4, and 8, and the number of individuals per population was 25. For DPART, *λ *was 1 for all loci and *α *was set such that the expected K value was equal to the true K value. Furthermore, we analyzed the data sets using only the Gibbs sampler by DPART for comparison. Although both DPART with the Gibbs and SAMS samplers and STRUCTURAMA were superior to Gibbs sampling alone, the former provided more accurate results than the latter, and the difference became prominent as K increased (Table 3). These results showed that the SAMS sampler was effective in population structure analysis. Note that the SAMS sampler has an additional advantage with regard to calculation time because one attempt of this sampler is faster than one scan with the Gibbs sampler, and unlike MCMCMC, the SAMS sampler does not need multiple chains.

**Table 3:** Evaluation of the SAMS sampler

		K = 2		K = 4		K = 8	
		
Program	algorithm	Microsatellite	SNP	Microsatellite	SNP	Microsatellite	SNP
DPART	Gibbs	0.016 (98)	0.292 (42)	0.228 (26)	0.476 (4)	0.315 (3)	0.523 (0)
		*2.06*	*1.42*	*3.15*	*2.12*	*5.74*	*3.93*
	Gibbs + SAMS	0.006 (100)	0.005 (100)	0.023 (97)	0.020 (97)	0.047 (89)	0.088 (68)
		*2.08*	*2.01*	*3.98*	*3.97*	*7.91*	*7.46*
STRUC-	Gibbs + MC^3^	0.006 (100)	0.025 (96)	0.039 (91)	0.118 (67)	0.122 (52)	0.264 (20)
TURAMA		*2.06*	*1.97*	*3.90*	*3.57*	*7.31*	*6.09*

Average , which is the partition distance between the true and inferred partition, the number of data sets in which  was 0.1 or less (in parentheses) in 100 simulated data sets, and the average K values in the inferred partitions (in Italic) are shown. The number of individuals in each population was 25. The number of loci was 20 for microsatellites and 100 and for SNPs. The migration rate was 0.003 for microsatellites. MC^3 ^indicates MCMCMC.

### Effect of *λ*

In the DP prior method, allele frequencies of populations are assumed to be drawn from the Dirichlet distribution *Dirichlet *(*λ*,*...*,*λ*). Because the variance of the distribution decreases as *λ *increases, the frequencies approach uniformity among alleles as *λ *increases. Thus, it was expected that *λ *would affect clustering behavior depending on the uniformity of frequencies among alleles, i.e., the preferable values of *λ *would vary depending on uniformity. We examined this hypothesis by analyzing data sets that were generated with simulation method 2. In this simulation method, the level of uniformity of frequencies among alleles could be determined by varying ancestral allele frequencies. Three scenarios were used for both microsatellites and SNPs. In one scenario, frequencies among alleles were relatively uniform at all loci (ancestral allele frequencies were {0.2,0.2,...,0.2} for microsatellites and {0.5,0.5} for SNPs). In another scenario, frequencies were skewed at all loci ({0.8,0.05,...,0.05} for microsatellites and {0.8,0.2} for SNPs), and in the last scenario, frequencies were relatively uniform for half of the loci and skewed for the other half. We analyzed these data sets with DPART using the Gibbs and SAMS samplers under different settings of *λ*.

As expected, the results showed that the preferable values of *λ *varied depending on the uniformity of frequencies among alleles (Tables 4 and 5). When the frequencies were closer to uniformity, higher values were preferable. In addition, when a data set included loci that differed significantly in the uniformity of frequencies among alleles, analysis with a single *λ *value was less accurate than that with inferring *λ *for each locus. These results suggest that to maximize the performance of the DP prior method, the *λ *value should to be determined properly for each locus according to the allele frequencies. Although inferring *λ *for each locus is a solution for this problem, the specification of *λ *in this manner tended to make inferences less accurate than assuming single values for all loci when the uniformities of allele frequencies were relatively equal among loci. Thus, we recommend that analyses be repeated under two different assumptions, a single *λ *value for all loci and a unique value for each locus. In each assumption, inferring *λ *would be useful.

**Table 4:** Effect of *λ *on the behavior of DPART (number of alleles was 5)

Ancestral allele freq. and number of loci	{0.2, 0.2 ..., 0.2} × 30 loci	{0.8, 0.05, ..., 0.05} × 100 loci	{0.2,0.2 ...} × 30 loci + {0.8, 0.05, ...} × 30 loci
Mean major allele frequency	0.353 ± 0.074	0.799 ± 0.105	0.575 ± 0.242

*λ *= 3	0.028 (97)	0.500 (0)	0.500 (0)
	*2.17*	*1.00*	*01.00*
*λ *= 1	0.044 (91)	0.500 (0)	0.236 (53)
	*2.65*	*1.00*	*1.53*
*λ *= 0.5	0.130 (75)	0.215 (57)	0.136 (73)
	*2.13*	*1.57*	*1.73*
*λ *= *J*_*l*_^-^^1^	0.459 (8)	0.020 (96)	0.370 (26)
	*1.18*	*1.96*	*1.26*
Inferred (unique)	0.034 (93)	0.475 (5)	0.028 (95)
	*2.24*	*1.05*	*1.95*
Inferred (single)	0.024 (99)	0.120 (76)	0.166 (67)
	*2.07*	*1.76*	*1.67*

Average , the number of data sets in which  was 0.1 or less (in parentheses), and the average K values (in Italic) are shown. The number of populations was 2 and the number of individuals per population was 25. Vectors in parentheses indicate ancestral allele frequencies. "Mean major allele frequency" indicates the mean values of major allele frequencies in the data sets. *J*_*l *_is the number of observed alleles. "Inferred (unique)" indicates that a unique *λ *value was inferred for each locus, and "Inferred (single)" indicates that a single value was inferred for all loci.

**Table 5:** Effect of *λ *on the behavior of DPART (number of alleles was 2)

Ancestral allele freq. and number of loci	{0.5, 0.5} × 50 loci	{0.8, 0.2} × 200 loci	{0.5, 0.5} × 50 loci + {0.8, 0.2} × 50 loci
Mean major allele frequency	0.621 ± 0.088	0.802 ± 0.114	0.711 ± 0.137

*λ *= 6	0.067 (83)	0.500 (0)	0.440 (12)
	*2.09*	*1.00*	*1.12*
*λ *= 3	0.085 (73)	0.470 (6)	0.176 (66)
	*2.31*	*1.06*	*1.66*
*λ *=	0.325 (36)	0.050 (90)	0.208 (59)
	*1.44*	*1.90*	*1.59*
Inferred (unique)	0.104 (70)	0.080 (84)	0.063 (89)
	*2.22*	*1.84*	*1.89*
Inferred (single)	0.068 (86)	0.035 (93)	0.073 (87)
	*2.02*	*1.93*	*1.87*

The number of populations was 2 and the number of individuals per population was 25.

### Analysis of the chicken data set

This data set, representing 600 chickens of 20 European breeds, was analyzed previously using STRUCTURE and HWLER [44,18]. Rosenberg *et al. *[44] examined K values ranging from 1 to 23 using STRUCTURE and selected 19 as the proposed K value according to likelihood. Then, 100 runs of STRUCTURE were performed assuming that K was 19. The authors reported that most breeds could be distinguished from each other, but breeds 44 and 45 shared a cluster in all runs. Pella and Masuda [18] analyzed the data set with HWLER assuming that K was 1 and detected 23 clusters. Similar to that in STRUCTURE analysis, breeds 44 and 45 were not distinguished. HWLER divided breed 21 into two clusters that included 14 and 16 individuals and detected three additional clusters that included 1, 1, and 3 individuals in breed 102. The three individuals in breed 102 were sampled from a flock of zoo animals, which were reported to be frequently assigned incorrectly in STRUCTURE analyses.

If the *λ *value is determined according to the number of alleles at each locus, similar to that in HWLER, *λ *ranges from 0.024 to 0.5 and the mean value is 0.138 because the number of alleles at each locus ranges from 2 to 41. The average major allele frequency across all loci and breeds was 0.631, indicating that the allele frequencies were relatively skewed. We analyzed this data set with DPART using the Gibbs and SAMS samplers, varying the *λ *value to demonstrate the extent to which *λ *affects the behavior of the DP prior method. *α *was set to 0.01, resulting in the expected K value of 1.06. The results are summarized in Table 6. When a unique *λ *value was inferred for each locus and *λ *was 0.05, the result was the same as that obtained with HWLER. The *λ *value inferred for each locus ranged from 0.040 to 0.579 and the average across loci was 0.131. On the other hand, when a single value was inferred for all loci, breed 21 was not divided into two clusters. The *λ *value inferred for all loci was 0.082. As *λ *was increased from 0.05 to 3, the number of detected clusters decreased. Thus, the finest partition was obtained when *λ *was , inferred for each locus, and set to 0.05. This indicates that for this data set, the specification of *λ *according to the number of alleles was appropriate. This is probably because of the fact that the allele frequencies at any loci were relatively skewed. These results also indicate that inferring *λ *was actually useful in empirical data sets.

**Table 6:** Summary of results for the chicken data set, representing 20 breeds

Program	*λ*	Number of clusters	Differences from the partition that was determined from breeds
HWLER		23	Breed 21 was divided into two clusters (14 and 16 individuals), breed 121 was divided into four clusters (1, 1, 3, and 25 individuals), and breeds 44 and 45 shared a cluster.
DPART	Inferred (unique)	23	Same as HWLER
	Inferred (single)	22	Breed 121 was divided into four clusters (1, 1, 3, and 25 individuals). Breeds 44 and 45 shared a cluster.
	0.05	23	Same as HWLER
	0.5	20	Breed 121 was divided into two clusters (5 and 25 individuals), breeds 44 and 45 shared a cluster, an individual in breed 5 shared a cluster with breed 50, and an individual in breed 16 shared a cluster with breed 5.
	1	17	Breeds 5 and 6, 18 and 37, and 44 and 45 shared different clusters respectively. Three individuals in breed 102 shared a cluster with breed 33. An individual in breed 5 shared a cluster with breed 50.
	3	9	Breeds 5, 16, 18, 21, 37 and 3402 shared a cluster. Breeds 33, 44, 45, 51 and an individual in breed 102 shared a cluster. Breed 13, 26, 42, 50, and an individual each in breeds 5 and 102 shared a cluster.

### Comparison among DPART, FUM, and FCM

We compared DPART, FUM, and FCM, focusing on cases with unbalanced sample sizes among populations. Data sets were generated by simulation method 1. First, we assumed situations in which K = 2, the size of the smaller subset was fixed at 10, the number of microsatellites was 20, and the migration rate was 0.003. The size of the larger subset was 10, 100, 200, and 300. Hereafter, the sample size is denoted as N (10, 10), N (10, 100), and so on. DPART was used with the Gibbs and SAMS samplers. The true K value was used for FUM and FCM. The results showed that DPART was insensitive to unbalanced sample sizes (Table 7). In contrast, FCM was the most sensitive of the three programs to unbalance. FUM was less sensitive than FCM, but was inferior to DPART when the sizes were N (10, 300). The difference among methods was most prominent at N (10, 300), but it decreased or disappeared when the migration rate decreased or the number of loci increased.

**Table 7:** Comparison among DPART, FUM, and FCM in data sets with unbalanced sample sizes

	Nl = 20					Nl = 50
	
	M = 0.003				M = 0.001	M = 0.003
	
	N (10, 10)	N (10, 100)	N (10, 200)	N (10, 300)	N (10, 300)	N (10, 300)
DPART	0.056 (83)	0.018 (95)	0.025 (94)	0.023 (96)	0.001 (100)	0.002 (100)
	*2.42*	*2.07*	*2.10*	*2.05*	*2.19*	*2.23*
FUM	0.024 (96)	0.010 (100)	0.009 (99)	0.095 (54)	0.001 (100)	0.001 (100)
FCM	0.053 (89)	0.041 (83)	0.146 (10)	0.190 (0)	0.024 (84)	0.021 (90)

Average , the number of data sets in which  was 0.1 or less (in parentheses), and the average K values (in Italic) are shown. Nl and M indicate the number of loci and the migration rate, respectively. N ( ) denotes sample sizes.

Next, we increased the size of the smaller subset to 50 to create a moderate unbalance. The number of loci was decreased to 10 and the migration rate was increased to 0.005 in order to compare the differences more clearly. Again, FCM was found to be most sensitive to unbalance (Table 8). Although the performance of DPART decreased slightly as the sample size became increasingly unbalanced, DPART provided the highest number of data sets in which  was 0.1 or less at N (50, 300). The difference among methods decreased or disappeared when the migration rate decreased or the number of loci increased.

**Table 8:** Comparison among DPART, FUM, and FCM in data sets with moderately unbalanced sample sizes

	Nl = 10					Nl = 20
	
	M = 0.005				M = 0.003	M = 0.005
	
	N (50, 50)	N (50, 100)	N (50, 200)	N (50, 300)	N (50, 300)	N (50, 300)
DPART	0.125 (73)	0.120 (66)	0.100 (62)	0.119 (52)	0.030 (99)	0.023 (100)
	*1.92*	*2.01*	*2.05*	*2.03*	*2.15*	*2.12*
FUM	0.073 (86)	0.072 (85)	0.081 (70)	0.118 (33)	0.023 (99)	0.016 (100)
FCM	0.074 (84)	0.078 (80)	0.103 (50)	0.146 (11)	0.039 (89)	0.023 (99)

Furthermore, we examined whether the number of minor subsets affected performance of these methods. We compared the methods in situations in which the sample sizes were N (10, 10, 200), N (10, 200, 200), N (50, 50, 200), and N (50, 200, 200). The migration rate was 0.003 in each situation. When multiple minor subsets were included in the data sets, i.e., at N (10, 10, 200) and N (50, 50, 200), DPART outperformed FUM and FCM, suggesting that FUM and FCM were severely affected by multiple minor subsets (Table 9). In contrast, when only one minor subset was included in the data set, i.e., at N (10, 200, 200) and N (50, 200, 200), the effect of the minor subset was relatively small.

**Table 9:** Comparison among DPART, FUM, and FCM in data sets with multiple small subsets

	Nl = 20		Nl = 50	Nl = 10		Nl = 20
	
	N (10, 10, 200)	N (10, 200, 200)	N (10, 10, 200)	N (50, 50, 200)	N (50, 200, 200)	N (50, 50, 200)
DPART	0.112 (66)	0.032 (92)	0.004 (100)	0.043 (99)	0.040 (99)	0.006 (100)
	*2.88*	*3.07*	*3.16*	*3.05*	*3.11*	*3.11*
FUM	0.435 (7)	0.012 (99)	0.334 (0)	0.154 (74)	0.035 (100)	0.057 (89)
FCM	0.496 (0)	0.055 (83)	0.364 (17)	0.162 (73)	0.041 (100)	0.072 (86)

The migration rate was 0.003

In these analyses, FUM and FCM often assigned individuals to clusters such that the sizes of the clusters were uniform, resulting in the failure of analysis. For example, at K = 2 or K = 3 with single minor subsets, the smaller subsets tended to absorb members in the larger subsets. At K = 3 with multiple minor subsets, FUM and FCM often failed to distinguish the two smaller subsets and divided the larger subset into three clusters such that their sizes were uniform. When the sizes were N (10, 10, 200) and the number of loci was 50, FUM frequently generated an empty cluster. In such cases, FUM detected only two clusters, consisting of two smaller subsets and the larger subset. Empty clusters were not observed when the number of loci was 20. Because the larger subset had no population structure, FUM probably detected the larger subset correctly as one cluster because of the increase in the number of loci. However, since FUM failed to divide the smaller subsets, only two clusters were detected by FUM. We did not observe this phenomenon in analyses with FCM; the difference in performance between FUM and FCM may be relevant in this situation.

### Analysis of the bull data set

**Figure 1:**
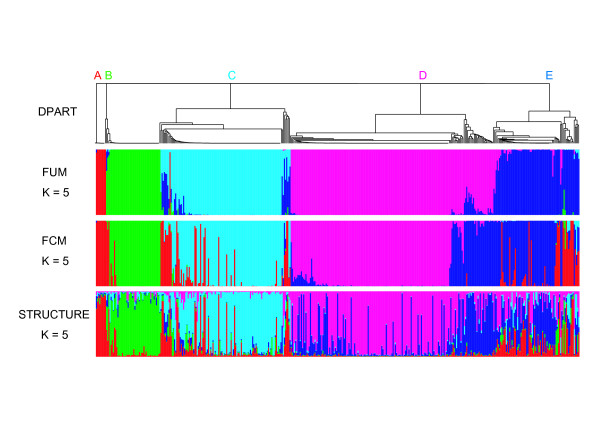
**Results obtained with each program during analysis of the bull data set The dendrogram of DPART was generated based on co-assignment probabilities for all individual pairs**. Each vertical bar in results for FUM and FCM represents the probability that the individual was derived from each population indicated by five colors. Each bar in the results of STRUCTURE represents the proportion of the individual's genome from each ancestral population. The bar plots were drawn by R [40]. The five clusters detected by DPART are referred to as clusters A, B, C, D, and E.

The bull data set, representing 427 bulls genotyped with 31 microsatellites, was analyzed by DPART, FUM, FCM, and STRUCTURE to demonstrate how unbalanced sample sizes among populations affect the results of these methods. In DPART, the *α *value was 0.5, resulting in the expected K value of 4. A single value of *λ *was inferred for all loci. As a result, we obtained two partitions, a partition with five clusters denoted as clusters A to E and a partition with four clusters. When a unique *λ *value was inferred for each locus, only the partition with four clusters was obtained. In this partition, cluster C was absorbed in clusters D and E. Thus, we created three data sets, each including clusters C and D, clusters C and E, or clusters D and E, and reanalyzed them with DPART. *λ *was inferred for each locus. Because clusters in each data set differed from each other, we concluded that the bull data set included five clusters. The dendrogram was generated on the basis of co-assignment probabilities calculated from 4,000 MCMC samples (Figure 1).

The data set was also analyzed using FUM, FCM, and STRUCTURE with K = 5. As shown in Figure 1, although the result obtained with FUM was consistent with that obtained with DPART (partition distance = 0.0328), the results obtained with FCM and STRUCTURE were not consistent with those obtained with FUM and DPART (partition distance = 0.2037 between DPART and FCM and 0.2881 between DPART and STRUCTURE). In the results of FCM and STRUCTURE, the smallest cluster in the results of DPART and FUM, i.e., cluster A (N = 9), absorbed the members of clusters B (N = 48), C (N = 115), and E (N = 76). In addition, the moderate cluster, cluster E, also absorbed the members of cluster D (N = 179). On the other hand, the second smallest cluster in the results of DPART and FUM, cluster B, absorbed very few members of the larger clusters in the results of FCM and STRUCTURE. The pairwise *F*_*st *_values between the clusters detected by DPART are shown in Table 10. We consider the interpretation of these inconsistent results in the Discussion section.

**Table 10:** Pairwise *F*_*st *_between clusters detected by DPART in the bull data set

	B	C	D	E
A	0.1085	0.0913	0.1495	0.0839
B		0.0759	0.1792	0.1024
C			0.1145	0.0548
D				0.0324

## Discussion

The Bayesian method based on the DP prior can infer the number of populations (K) and assign individuals, whereas the selection of the appropriate K value is often problematic when methods that run under a predefined K value are used. We examined the properties of this method to provide information that will be useful in practice. We showed that the SAMS sampler, which assigns multiple individuals simultaneously, was effective for the inference of population structures. Because SAMS sampling was faster than MCMCMC techniques, the implementation of this sampling technique may be especially useful for large data sets. We also showed that a hyperparameter, named *λ*, which defines the prior distribution of allele frequencies, affected the performance of the method and its specification was important. This problem could be resolved by considering *λ *a variable. Furthermore, we demonstrated that the DP prior method was suitable for data sets having unbalanced sample sizes among populations, whereas methods that implement STRUCTURE-like algorithms were sensitive to unbalance. In particular, we found that the allele frequencies correlated model was the most sensitive.

Our results showed that both the SAMS sampler and MCMCMC were effective in improving the mixing of MCMC algorithms; however, the SAMS sampler was more effective than MCMCMC. We implemented the SAMS sampler at a frequency in which four iterations with the SAMS sampler were followed by one iteration with the Gibbs sampler (four SAMS and one Gibbs). Although we examined other frequencies, such as two SAMS and five Gibbs, one SAMS and one Gibbs, and five SAMS and two Gibbs, the results were almost the same (data not shown). We selected this frequency simply to shorten the run time, because one attempt of the SAMS sampler is faster than one scan of the Gibbs sampler. However, an accurate inference of the posterior distribution is hardly possible with the SAMS sampler alone and the Gibbs sampler is necessary. The acceptance rate of the proposed states was extremely low (typically below the order of 10^-3 ^for both split and merge), suggesting the difficulty of proposing new partitions. STRUCTURAMA implementing MCMCMC can probably increase its performance by increasing the number of chains or adjusting the temperature parameter to obtain the appropriate exchange rates among chains. However, calculation time increases as the number of chains increases and multiple runs may have to be performed to find appropriate values for the temperature parameter. Therefore, we concluded that the SAMS sampler was more useful in practice and implemented this sampler in DPART to improve the mixing of MCMC algorithms.

We showed that the choice of *λ *values affected the behavior of the DP prior method in the simulated and real data sets. Since the preferable *λ *value varies depending on the uniformity of frequencies among alleles, it is desirable that the *λ *value at each locus is determined according to the allele frequencies. Because HWLER set *λ *to  for any loci, its performance is probably inadequate for some data sets, although it implements the SAMS sampler. STRUCTURAMA does not state how it specifies *λ*, but it appears to fix *λ *for any loci at a certain value. Inferring a unique *λ *value for each locus is a method of specifying the parameter for each locus. However, this approach resulted in less accurate inferences than assuming a single value for all loci when the levels of uniformity of frequencies among alleles were relatively similar among loci. We speculate that increasing the number of hyperparameters to be estimated may make inferences unstable. In contrast, assuming a single value for all loci was less accurate in data sets in which the levels of uniformity differed significantly among loci. Therefore, it is difficult to state which assumption is more suitable for the data set of interest. In the chicken data set, the unique value assumption (*λ *is inferred for each locus or *λ *is ) and the single value assumption (*λ *= 0.05) provided the same partition. However, analysis in which a single value was assumed and inferred for all loci gave a slightly rougher partition. In the bull data set, the single value assumption (a single *λ *value is inferred for all loci) gave a finer partition than the unique value assumption (*λ *is inferred for each locus). In general, if the loci included in the data have not been evaluated well with regard to polymorphism, some loci may be much less polymorphic than others, and thus, the allele frequencies at these loci will be skewed. For such data sets, the unique value assumption will be suitable. On the other hand, if the loci included in the data are selected from a large number of candidate loci, they will be highly polymorphic, and thus, the allele frequencies at these loci are expected to be close to uniformity. Therefore, the single value assumption will be suitable for such data sets. We speculate that the chicken data may be closer to the former case and, in such a case, even if an appropriate single value that leads to an accurate inference exists, the inference of such a value may be difficult. In contrast, the bull data set may be closer to the latter case because the loci included in the data set had been selected from a large number of candidates for parentage testing. Therefore, the single value assumption may be preferable.

Our results showed that the behavior of the DP prior method depends on the selection of *λ*. We speculated that integration out of allele frequencies involves this dependency to some extent. Thus, although we have not examined this speculation, the dependency may decrease by inferring allele frequencies. However, this will increase the calculation time and thus will not be suitable for large data sets.

We found that unbalanced sample sizes among populations affect the behavior of DPART, FUM, and FCM. FUM and FCM were found to be sensitive to unbalanced sample sizes, and their performances were substantially affected, particularly by the presence of multiple minor subsets. The reason why DPART is suitable for unbalanced sample sizes is probably its prior assumptions about the assignment of individuals. As seen in Eq. (3), in the algorithm implementing the DP prior, clusters absorb individuals with higher probability as sample sizes increase, i.e., the "rich get richer" phenomenon occurs. Thus, this algorithm is suitable for data sets with unbalanced sample sizes among populations. On the other hand, the algorithms implemented in FUM, FCM, and STRUCTURE assume that each population can contribute to the data set with equal probability. Thus, these algorithms are suitable for data sets in which sample sizes are uniform among populations. When these methods are used for data sets with unbalanced sample sizes, they tend to cluster individuals such that the sizes become uniform among clusters, as observed in our experiments. The extent of sensitivity varied depending on the number of loci and the migration rates when the level of unbalance was fixed. Thus, if genetic differences among populations are small and sample sizes are unbalanced, then the number of loci needed by these methods to correctly detect population structures is higher than that needed by DPART. Although we compared these programs using only microsatellite data, the differences in behavior among them will not vary if SNP data are analyzed because the differences will be due to differences among the prior assumptions and will not depend on the number of alleles.

The sensitivity of FUM and FCM to unbalance can probably be resolved by adding parameters that represent the mixing weights to the algorithms. As described by Neal [32], when the prior distribution of the mixing weights is assumed to be a Dirichlet distribution such as  where *K *is the assumed number of populations and these weights are integrated out, we can obtain a predictive distribution for the assignment of the *i*th individual,

which is similar to Eq. (3), but now in the parametric framework. FUM, FCM, and other STRUCTURE-like algorithms will be able to deal with unbalanced sample sizes by implementing this prior distribution.

FCM, which implements the allele frequency correlated model, was shown to be more sensitive than the other programs to unbalanced sample sizes. A refutation of this may be that comparison among methods was performed for simulated data sets generated using the isolated population models that do not accord with the assumption of FCM. However, FCM was most sensitive to unbalance in the data sets that were generated by simulation method 2, which was based on the assumption of FCM (data not shown). We speculated that correlated allele frequencies are involved in the sensitivity of FCM. In the allele frequencies correlated model, the conditional posterior distribution for allele frequencies of population *j *is given as follows:

where *y*_*jlh *_is the number of copies of allele *h *at locus *l *in individuals assigned to population *j*, *Pa*_*lh *_is the ancestral frequency of allele *h *at locus *l*, *J*_*l *_is the number of alleles at locus *l*, and *f*_*j *_is  where *F*_*j *_is the drift parameter for population *j*. Thus, as the sample size for a population decreases, the effect of ancestral allele frequencies on the inference of allele frequencies for this population increases. On the other hand, ancestral allele frequencies are inferred from information on allele frequencies of populations and drift parameters. Therefore, if unbalanced sample sizes are present, ancestral allele frequencies will be affected more strongly by the inferred allele frequencies for major subsets because those for minor subsets are substantially affected by ancestral allele frequencies themselves, and are thus, less informative for the inference of ancestral allele frequencies. Consequently, the inferred allele frequencies for minor subsets will be affected by those for major subsets and will approach them. Therefore, minor subsets may be prone to absorbing members of major subsets.

We interpreted the inconsistency found in the bull data set on the basis of the knowledge obtained from simulations. If the results obtained with DPART and FUM are correct, this inconsistency can be explained by the sensitivity to the unbalance of the allele frequencies correlated model. FCM and STRUCTURE probably failed to detect the smallest cluster (cluster A) because of their sensitivity. The failure of cluster A to absorb the members of cluster D in the results of FCM and STRUCTURE was due to the high level of differentiation between these clusters (pairwise *F*_*st *_= 0.1495 in Table 10). Although the unbalance between clusters D and E was moderate (N = 179 and 76), FCM and STRUCTURE also presumably failed to distinguish these clusters because of the relatively low pairwise *F*_*st *_between these clusters (0.0324). When the data set that included only clusters D and E was analyzed by FCM, this program also failed to distinguish the two clusters (data not shown). On the other hand, because cluster B was well differentiated from larger clusters (pairwise *F*_*st *_= 0.0759 between B and C, 0.1792 between B and D, and 0.1024 between B and E), every program was able to detect the cluster. The presence of multiple minor clusters was also considered to reduce the performance of FCM and STRUCTURE. In fact, when the two data sets that included only clusters A and B and clusters A and E were generated and analyzed by FCM, this program was able to distinguish the two clusters in each data set (data not shown). Although we admit that we have not proved that the results of DPART and FUM are correct, we believe that our interpretation is appropriate because it can clearly explain the inconsistency.

For the bull data set, 10 runs were performed with FCM and STRUCTURE. The results of the 10 runs of each program were almost similar, and we considered them to be incorrect. However, in the simulated data sets with unbalanced sample sizes among populations, we occasionally observed that these programs or FUM provided both correct and incorrect results when multiple runs were performed (data not shown). Thus, when the *ad hoc *statistic proposed by Evanno *et al*. [45] is used to select the true K value, this phenomenon possibly confuses the selection because it increases the standard deviation of likelihood at the true K value.

The effect of sample sizes of populations on the performance of clustering programs was addressed in some studies [[44,46] and [47]]. However, the effect of unbalanced sample sizes has been overlooked, and simulation studies for comparison of clustering methods usually assume uniform sample sizes among populations [12,48]. Because our results showed that sensitivity to unbalance in sizes varied among the methods, we recommend that comparative studies consider the effect of unbalance during analyses.

Through analyses of simulated data sets by DPART, we observed overestimation of K caused by small clusters that included only one or two individuals. This phenomenon increased the average K values and slightly affected the average . As discussed above, the DP prior method can efficiently detect minor subsets because of the "rich get richer" phenomenon. Thus, we speculated that the overestimation was due to the fact that DPART detected individuals that were slightly distanced from the other members because even in simulated data sets, individuals harboring rare genotypes can be generated with low probabilities. This interpretation was supported by phylogenetic analysis based on genetic distances between individuals (data not shown). In addition, this may be supported by the fact that overestimation became prominent as the number of loci, i.e., the power to detect population structures, increased (Tables 7, 8, and 9). Therefore, such small clusters are interpreted as overestimations in simulation studies, but will provide useful information in empirical studies because they indicate the presence of genetic discontinuity in the data sets.

## Conclusions

This study characterized the Bayesian method of implementing the DP prior and introduced a program, named DPART, in order to infer population structures more accurately than preceding programs based on the DP prior. First, we showed that the SAMS sampler, which is a technique for improving the mixing of MCMC algorithms, was effective for population structure analysis. Implementation of this sampler was useful with regard to the accuracy of inference and computing time. Second, we showed that a hyperparameter for the prior distribution of allele frequencies affected the behavior of the DP prior method. Appropriate values can be specified by inferring this parameter. Third, the DP prior method was shown to be suitable for analysis of data sets with unbalanced sample sizes among populations. In contrast, methods that implement STRUCTURE-like algorithms were shown to be suitable for data sets with uniform sample sizes among populations, but not for data sets with unbalanced sample sizes. Because these differences can yield inconsistent results among methods, we recommend using these methods concurrently. When the results obtained are inconsistent among methods, considering the effect of unbalanced sample sizes may be a key to interpreting the inconsistency.

## Competing interests

The authors declare that they have no competing interests.

## Authors' contributions

AO conceived the study, performed all statistical analyses, and drafted the manuscript. MN and MM collaborated on the algorithm design and the manuscript. All authors have read and approved the final manuscript.

## Endnote A - Gibbs sampler

One scan of the Gibbs sampler consists of the following steps.

Step 1. Remove the *i*th individual from *η*.

Step 2. Assign *i *to existing populations according to probabilities

where *1 *≤ *j *≤ *K*, or a new population according to probability

Step 3. Update *η*.

Step 4. Repeat Steps 1-3 for all *i *∈ {1,...,*n*}.

## Endnote B - SAMS sampler

The algorithm of the SAMS sampler is as follows.

Step 1. Select two individuals, *i *and *j*, at random.

Step 2. If *i *and *j *belong to the same population *S*, remove *i *and *j *from *S *and form two singletons, *S*_*i *_= {*i*} and *S*_*j *_= {*j*}. If not, proceed to Step 5.

Step 3. Assign the individuals remaining in *S *to *S*_*i *_or *S*_*j*_. The order of the assignment is randomly determined. The *k*th individual is assigned to *S*_*i *_with probability(7)

Otherwise, add the individual to *S*_*j*_.

Step 4. The proposed partition *η*' is accepted with MH probability(8)

where *q*(*η*'|*η*) and *q*(*η*|*η*') are the transition probabilities. Using Eq. (5), the ratio of likelihoods in Eq. (8) can be written as follows:

Using Eq. (4), the ratio of prior probabilities in Eq. (8) can be written as follows:(9)

*q*(*η*|*η*') is 1 and *q*(*η*'|*η*) results from the products of probabilities of the assignment given in Eq. (7).

Step 5. If *i *and *j *belong to different populations, say *S*_*i *_and *S*_*j*_, propose a new population *S *by merging *S*_*i *_and *S*_*j*_. The proposed partition *η*' is accepted with the probability given in Eq. (8). The ratio of likelihoods can be written as follows:

and the ratio of prior probabilities is given as follows:

*q*(*η*'|*η*) is 1 and *q*(*η*|*η*') is computed by splitting *S *into *S*_*i *_and *S*_*j *_in a randomly determined order.

## Endnote C - prior distribution of K

Prior distribution for K can be inferred using the following Monte Carlo procedure.

Step 1. Let the first individual belong to the first population and let K = 1.

Step 2. Assign individual *i *= {2,3,...,*n*} to existing or new populations with the probabilities noted in Eq. (3).

Step 3. Record K after the assignment of the last individual.

Step 4. Repeat Steps 1-3 for sufficient cycles to infer the distribution of K (e.g., 10,000).

## Endnote D - mean partition

The algorithm for calculating the mean partition described by Huelsenbeck and Andolfatto [19] is as follows.

Step 1. Pick a sampled partition as the initial state of the mean partition and calculate *D*, which is the sum of the partition distances between the mean partition and every sampled partition.

Step 2. Pick an individual *i *in the mean partition. Propose new mean partitions by moving *i *to other populations in the mean partition and to a new partition. Calculate the sum of partition distances between each proposed mean partition and each sampled partition, which is denoted as *D*'.

Step 3. Let *D*'_min _denote the minimum value of *D*'*s*. If *D*'_min _<*D*, the corresponding proposed mean partition is accepted and *D *is replaced by *D*'_min_.

Step 4. Repeat Step 2 and 3 for *i *= {1,2,...,*n*}.

Step 5. Repeat Steps 2, 3, and 4 until *D *stops decreasing.

## Supplementary Material

Additional file 1 **An R function for agglomerative hierarchical clustering**. This performs agglomerative hierarchical clustering based on the results of DPART. Click here for file

Additional file 2 **Executable file of DPART**. This file will work on a Windows platform. Click here for file

Additional file 3 **Parameters**. This file defines the parameters for DPART. Click here for file

Additional file 4 **Input file names**. This file defines the input file names for DPART. Click here for file

Additional file 5 **Source code**. This is the source code of DPART. Click here for file

Additional file 6 **Header file**. This is the header file of DPART. Click here for file

Additional file 7 **Manual**. This is the manual for DPART. Click here for file

Additional file 8 **Bull data set**. This file contains the genotypes of the bulls used in this study. This file is also an example of input data for DPART. Click here for file
