# Supplementary material for: Characterization of a Bayesian genetic clustering algorithm based on a Dirichlet process prior and comparison among Bayesian clustering methods
Source: BMC Bioinformatics. 2011 Jun 28;12:263. doi: 10.1186/1471-2105-12-263 (PMC3161044; doi:10.1186/1471-2105-12-263)
Supplement: Additional file 7 — Manual. This is the manual for DPART. [file 1471-2105-12-263-S7.ZIP › Additional file 7/Manual for DPART.pdf]

Manual for DPART

March, 2011

Akio Onogi (onogi@liaj.or.jp)

[Data formats of input genotype files]

Data for each individual are stored in one row. If a data set consists of two diploid individuals and three microsatellites, the format is as follows.

123 125 94 96 240 242
121 125 90 94 -9 -9

The labels of alleles should be integers between 1 and 999. "-9" represents the missing data. If a data set consists of two haploid individuals and three microsatellites, the format is as follows.

118 168 120
120 166 120

Any other information than genotypes (e.g. labels of individuals or names of loci) should not be included. "Bulldata.txt" is an example of input genotype files. This contains 427 diploid individuals genotyped with 31 microsatellites.

[Specification of the input genotype files to be analyzed]

The input genotype files to be analyzed are specified in the file "DPART_inputfiles.txt" as follows.

Input1.txt
Input2.txt
Input3.txt

Here, the number of input genotype files is three. These files are analyzed with common parameter values that are defined in the file "DPART_parameters.txt" as described below. The length of the input file names is up to 50 letters.

[Parameters]

Parameters below are defined in the file “DPART_parameters.txt”.

Ninput:	Number of input genotype files to be analyzed. The names of these files are specified in “DPART_inputfiles.txt”.
Ploidy:	Haploid (1) or diploid (2).
Ni:	Number of individuals.
Nl:	Number of loci.
Alpha:	Concentration parameter of Dirichlet process. This parameter and the number of individuals define the expected number of populations. If *Alpha* = 0, this parameter is inferred. Otherwise, the parameter is set to the given value. See [Appendix] to choose *Alpha*. *Alpha* should be > 0. Inferring *Alpha* and *Lambda* simultaneously is not recommended.
Lambda	Hyperparameter that defines the prior distribution of allele frequencies. When *Lambda* = 0, a unique value of *Lambda* is inferred for each locus. When *Lambda* = -1, a single value of *Lambda* is inferred for all loci. We recommend that analyses be repeated under these two assumptions. When *Lambda* = -2, *Lambda* for each locus is set to 1/*Jl* where *Jl* is the number of allele at locus *l*. When *Lambda* > 0, *Lambda* for all loci is set to the given value.
Burnin:	Length of burn-in.
Iterations:	Length of iterations after burn-in.
Thinning:	Interval of MCMC sampling.<br>The number of MCMC samples is *Iterations*/*Thining*.
Run:	Number of runs.
Meanpartcal:	Indicates whether the mean partition is calculated.<br>1 (yes) or 0 (no). If a data set includes a large number of individuals (e.g., 1000) or consists of a large number of populations (e.g., 20), the calculation of mean partitions takes time.

Parameters below are defined in the source code. Modifications may not be needed.

seed (-1):	Random seed. When -1, time is used.
SAMSfreq (4):	Frequency of the SAMS sampler. If *SAMSfreq* = 0, the SAMS sampler is not used.
Gibbsfreq (1):	Frequency of the Gibbs sampler. When *SAMSfreq* = 4 and *Gibbsfreq* = 1, four iterations with the SAMS sampler are followed by one iteration with the Gibbs sampler.
Shape (1.0):	Shape of the gamma prior distribution of Alpha. Only used when *Alpha* = 0.
Scale (1.0):	Scale of the gamma prior distribution of Alpha. Only used when *Alpha* = 0. E [Alpha] = Shape/Scale.
Sdforalpha (0.02):	Standard deviation of the normal proposal distribution for Alpha. Only used when *Alpha* = 0.
Lambdalim (10.0):	The prior distribution of *Lambda* is Uniform (0, *Lambdalim*). Only used when *Lambda* = 0 or -1.
Sdforlambda (0.02):	Standard deviation of the normal proposal distribution for *Lambda*. Only used when *Lambda* = 0 or -1.

[Running DPART]

Put input genotype files, “DPART_inputfiles.txt”, “DPART_parameters.txt”, and the executable file (DPART.exe) in the same directory and start the executable file. The executable file that we provide can work on Windows.

[Output files]

DPART outputs files below.

Input genotype file name_parameters.txt:

Used parameter values.

Input genotype file name_coaprob_runX.txt:

Co-assignment probabilities for all individual pairs.

An example is as follows (number of individuals is four).

co-assignment probabilities

Individual numbers and corresponding probability

1 2 0.988

1 3 0.214

1 4 0.112

2 3 0.153

2 4 0.002

3 4 0.897

The individual numbers correspond to the row numbers of the input genotype file. “1 2 0.988” indicates that the co-assignment probability between individual 1 and 2 is 0.988.

Input genotype file name_Z_runX.txt:

Sampled partitions during MCMC iterations.

Input genotype file name_Acrate_runX.txt (when *SAMSfreq* > 0):

Acceptance rates and frequencies of the SAMS sampler at each window. A window consists of (*Burnin* + *Iterations*)/10 iterations. “freq” and “rate” indicate the frequencies and rates, respectively. Acceptance rates of the SAMS sampler are usually low.

Input genotype file name_meanpartition_runX.txt (when *Meanpartcal* = 1):

Mean partition calculated from “Input genotype file name _Z_runX.txt”.

Input genotype file name_meanLambda_runX.txt (when *Lambda* = 0 or -1):

Average *Lambda*.

Input genotype file name_Lambda_runX.txt (when *Lambda* = -2):

Values of *Lambda* that are determined based on the numbers of alleles.

Input genotype file name_meanAlpha_runX.txt (when *Alpha* = 0):

Average *Alpha*.

[Processing the output file]

Agglomerative hierarchical clustering can be performed from the co-assignment probabilities. We use the hclust function of the stats package of R. For R, see http://cran.r-project.org/ . “procdpart.R” is an example function for the clustering. After importing the “procdpart.R” file to R and changing the working directory of R to the directory where the “Input genotype file name_coaprob_runX.txt file” is put, clustering can be performed by typing

procdpart (“Input genotype file name_coaprob_runX.txt”, *Ni,* Nc, Needlabels)

in the R console. The tree is generated in pdf format. This function can cut the tree into “Nc” clusters and output the labels of clusters that individuals belong to, as follows.

Indi.number	Clus.number
1	1
2	1
3	2
4	1
5	3

“Indi.number” indicates individual numbers (i.e., row numbers of input genotype file), and “Clus.number” indicates the labels of clusters that the corresponding individuals belong to. In this example, individuals 1, 2, and 4 are assigned to cluster 1, individual 3 is assigned to cluster 2, and individual 5 is assigned to cluster 3. “Needlabels” indicates whether individual numbers are drawn in the tree or not.

In addition, this function can output where individuals are located in the tree as follows.

From L to R	Indi.number
1	5
2	3
3	2
4	1
5	4

In this example, individual 5 is located in the leftmost in the tree and individual 4 is located in the rightmost.

This function can be arranged with WordPad or NotePad.

[Appendix]

Choose the value of *Alpha* from the table below based on the number of individuals and the expected number of populations. For example, if your data set includes 100 individuals and you expect that these individuals are derived from five populations, 0.94 is appropriate.

		Number of individuals														
		20	50	80	100	150	200	250	300	350	400	450	500	1000	1500	2000
Expected number of populations	2	0.32	0.24	0.22	0.20	0.19	0.18	0.17	0.17	0.16	0.16	0.16	0.15	0.14	0.13	0.13
	3	0.72	0.52	0.45	0.43	0.40	0.37	0.36	0.34	0.33	0.33	0.32	0.31	0.28	0.27	0.26
	4	1.20	0.83	0.72	0.68	0.62	0.58	0.55	0.53	0.52	0.51	0.50	0.49	0.43	0.41	0.40
	5	1.80	1.18	1.01	0.94	0.85	0.80	0.76	0.73	0.71	0.69	0.67	0.66	0.59	0.56	0.53
	6	2.54	1.56	1.32	1.24	1.10	1.03	0.98	0.94	0.91	0.88	0.87	0.84	0.75	0.71	0.68
	7	3.42	1.98	1.66	1.54	1.37	1.27	1.20	1.16	1.12	1.08	1.06	1.04	0.92	0.87	0.83
	8	4.47	2.46	2.01	1.87	1.65	1.52	1.44	1.39	1.34	1.30	1.26	1.24	1.09	1.02	0.97
	9	5.72	2.95	2.40	2.22	1.95	1.80	1.70	1.61	1.57	1.51	1.47	1.45	1.27	1.18	1.14
	10	7.31	3.49	2.81	2.58	2.24	2.08	1.94	1.85	1.79	1.74	1.69	1.66	1.45	1.36	1.29
	12		4.70	3.67	3.35	2.91	2.65	2.47	2.36	2.28	2.21	2.15	2.09	1.82	1.70	1.62
	14		6.10	4.67	4.20	3.61	3.27	3.05	2.90	2.79	2.70	2.62	2.55	2.21	2.04	1.94
	16		7.71	5.74	5.16	4.33	3.91	3.65	3.47	3.31	3.20	3.11	3.04	2.59	2.41	2.29
	18		9.63	6.92	6.14	5.15	4.61	4.30	4.03	3.87	3.75	3.62	3.54	3.00	2.79	2.63
	20			8.25	7.25	5.98	5.35	4.94	4.65	4.45	4.30	4.15	4.05	3.44	3.16	3.00
	30						9.59	8.70	8.11	7.68	7.37	7.08	6.85	5.72	5.20	4.92
	40													8.21	7.46	7.00
	50														9.83	9.19
